# Role of a common variant of Fat Mass and Obesity associated (*FTO*) gene in obesity and coronary artery disease in subjects from Punjab, Pakistan: a case control study

**DOI:** 10.1186/s12944-016-0200-0

**Published:** 2016-02-16

**Authors:** Saleem Ullah Shahid, Shabana, Abdul Rehman, Shahida Hasnain

**Affiliations:** Department of Microbiology and Molecular Genetics, University of the Punjab, Lahore, 54590 Pakistan; The Women University Multan, Multan, Pakistan

**Keywords:** FTO, Coronary artery disease, Obese

## Abstract

**Background:**

The *FTO* gene has recently become one of the most extensively investigated genes associated with body mass and has been shown to play a role in cardiovascular diseases as well. The aim of the current study was to investigate the effect of a common variant of *FTO* gene, rs9939609 in obese and coronary heart disease (CHD) patients of Pakistan and investigate whether it has any influence on the serum biochemical parameters.

**Methods:**

A total of 970 samples (295 obese, 425 CHD and 250 controls) were genotyped using TaqMan allelic discrimination assay. Serum total cholesterol, HDL-C and triglycerides were measured using spectrophotometric methods. LDL-C was calculated by Friedwalds equation. Statistical analysis was done by SPSS version 22.

**Results:**

Results showed moderately high minor allele frequency (MAF) in obese and CHD cases as compared to controls (obese = 0.381 CAD = 0.361 and controls = 0.286). The variant was significantly associated with obesity and CAD (obesity odds ratio (OR) = 1.54, confidence interval (CI) = 1.07–2.21, *p* = 0.0009; CHD OR = 1.43, CI = 1.02–2.01, *p* = 0.004) in Pakistan. The risk allele did not show a significant association with any of the lipid trait tested (*p* > 0.05) but a strong association was observed with plasma glucose levels (obese *p* = 0.001, CAD *p* = 0.014, controls *p* = 0.019).

**Conclusion:**

In conclusion, the variant was associated with obesity and CAD in the studied subjects and its possible effect may involve the blood sugar metabolism but not serum lipid chemistry.

**Electronic supplementary material:**

The online version of this article (doi:10.1186/s12944-016-0200-0) contains supplementary material, which is available to authorized users.

## Background

Obesity, defined as excessive accumulation of body fat, is a complex disorder whose pathogenesis involves both environmental and genetic factors. Obesity, a complex disorder, is a strong predisposing risk factor for several other diseases, like diabetes, hypertension and CAD [1]. In Pakistan a dramatic increase in the prevalence of obesity has been observed recently and currently 5.2 % females and 1.6 % males in Pakistan over the age of 15 are obese [2]. As the preventive and therapeutic measures employed so far have failed to bring about the expected results, new pathogenic mechanisms of the disease are being looked for. However, only a small proportion of body mass index (BMI) could be explained on genetic bases due to small effect size of the variants studied [3]. While some forms of obesity are caused by single mutations, most cases are polygenic and result from a complex interaction between the genotype and the environment [4].

Coronary heart disease (CAD) is the leading cause of death worldwide. Despite being manageable, the mortality rate is increasing in developing countries and the disease burden is likely to be doubled by 2020 in these countries [5]. Asian population is more susceptible to heart diseases and a 50 % higher prevalence of cardiovascular diseases has been reported in South Asians [6]. Pakistan which is a country of >180 million people has high burden of CAD like rest of the world [7]. The prevalence of CAD risk factors is high in Pakistani population and more than 30 % of the people above 45 years of age are affected by the disease [8]

CAD being a complex interplay between environmental, life style and genetic factors, a large number of genes have been investigated for having their role in the development of the disease. As CAD can be a sequel of obesity, the genes involved in the development of obesity have also been investigated of having any role in the development of CAD [9].

The *FTO* gene has recently become one of the most extensively investigated genes associated with body mass [10]. It spans more than 400Kb on chromosome 16 and has nine exons [11]. Despite large size of *FTO* gene, variants implicated in obesity and weight gain are largely located in the first intron [12]. FTO protein is a 2-oxyglutarate dependent non-heme dioxygenase family member and localizes in nucleus [13]. Previously *FTO* was thought to be highly expressed in the hypothalamic nuclei involved in energy balance but recent rodent studies suggest that in addition to hypothalamus, it is also expressed in the peripheral tissues like pancreas [14]. The association of *FTO* variant, rs9939609 with obesity has been confirmed in Caucasians, and the results in Asian populations were initially somewhat conflicting [15–21], however, substantial data on association of this variant with BMI and obesity is now available in Chinese, Japanese, Korean and Filipino populations [22–25]. A meta-analyses of published studies in Asians reported that the minor allele for the rs9939609 significantly (*p* = 9 × 10^−9^) increased the risk of obesity [10]. Fewer studies in South Asians have been reported, two of which confirmed the association between the *FTO* locus and obesity susceptibility [26, 27], whereas one study did not [28].

The association of *FTO* gene variant rs9939609 with CAD has been reported by many studies [29–31]. Though not fully understood, the underlying mechanism of progression to ischemic heart disease involves an array of events like insulin resistance, endothelial damage and inflammation [32]. Since obesity, metabolic syndrome and CAD are interrelated through a complex interaction of genetic and environmental factors [33], the SNP has also been reported to be associated with cardiovascular disease [34]. Keeping in view the importance of this variant in energy metabolism and contradictory results reported so far, we aimed to investigate the association of the *FTO* rs9939609 T > A single-nucleotide polymorphism (SNP) in Pakistani obese, CAD patients and control subjects and observe the effect of this variant on selected biochemical parameters.

## Results

The baseline characteristics of the study subjects are given in Table 1. A higher proportion of males were having CAD and obesity than females, but the difference was not significant. The CAD cases belonged to an elder age group as compared to controls (*p* = 0.002) whereas the age difference was not significant in obese cases and controls. The lipid profile parameters including TC, LDL-C and TG as well as blood sugar levels were significantly higher in obese and CAD cases as compared to controls. Whereas HDL-C was lower in both cases than control subjects. The genotype call rate was 97 % for obese, 98 % for CAD and 98 % for the control group. The mean weight, BMI and WHR for obese subjects were 96.16 ± 15.95, 36.92 ± 6.46 and 1.00 ± 0.09 respectively, for CHD subjects were 67.13 ± 11.95, 22.46 ± 6.75 and 0.81 ± 0.06 respectively and for control subjects 67.13 ± 9.56, 21.46 ± 9.11 and 0.85 ± 0.10 respectively. Overall, the study population was in Hardy-Weinberg equilibrium (*p* = 0.938).

**Table 1:** Baseline features of subjects under study

Characteristic	CAD			Obesity		
	Cases (*n* = 425)	Controls (*n* = 250)	*p*-value	Cases (*n* = 295)	Controls (*n* = 250)	*p*-value
Gender (M/F %)	59/41	54.3/45.7	0.271	56.2/43.8	54.3/45.7	0.873
Age (years)	59 ± 12	56 ± 10	0.002	55.32 ± 15.18	56.01 ± 10.44	0.31
TC (mmol/L)	5.36 ± 1.38	4.51 ± 1.11	9.1 × 10^-14^	5.54 ± 1.11	4.51 ± 1.11	2.0 × 10^-24^
TG (mmol/L)	2.43 ± 0.83	2.12 ± 0.74	3.6 × 10^-8^	2.39 ± 0.79	2.12 ± 0.74	3.1 × 10^-5^
HDL-C (mmol/L)	1.11 ± 0.11	1.74 ± 0.42	5.9 × 10^-7^	1.16 ± 0.31	1.74 ± 0.42	1.9 × 10^-66^
LDLD-C (mmol/L)	2.74 ± 0.74	2.18 ± 0.43	2.4 × 10^-5^	2.99 ± 0.56	2.18 ± 0.43	1.4 × 10^-21^
FBG (mg/dL)	98.42 ± 6.61	89.56 ± 7.29	1.0 × 10^-46^	102.71 ± 14.12	89.56 ± 7.29	2.4 × 10^-31^

The table summarizes the general characteristics of the study subjects by comparing the cases with controls. *CAD* Coronary heart Disease group, *TC* Total Cholesterol, *TG* Triglycerides, *HDL-C* High Density Lipoprotein cholesterol, *LDL-C* Low Density Lipoprotein cholesterol, *FBG* fasting blood glucose

### Association of FTO rs9939609 polymorphism with obesity and CAD

The allele and genotype frequencies of the rs9939609 variant are shown in Table 2. The minor allele frequency (MAFs) in CAD group was significantly higher than controls (0.361 vs 0.286. Similarly, the MAF was higher in obese cases than controls (0.381 vs 0.286). Genotype distribution revealed significant differences between the cases and controls (CAD: TT = 44 %, TA = 39.76 %, AA = 16.24 %; obese: TT = 39.32 %, TA = 45.08 %, AA = 15.59 % vs controls TT = 45.2 %, TA = 36.4 %, AA = 18.4 %). Overall, the frequency of TT genotype was 42.88 %, of TA was 40.51 % and of AA was 16.59 %. The *FTO* rs9939609 polymorphism was found to be significantly associated with obesity as well as CAD in Pakistani subjects. The per allele odds ratio (OR) for CAD was 1.43 (1.02–2.01) which is a significant association (*p* = 0.004). Similarly the association with obesity was found to be significant (*p* = 0.0009) with an OR of 1.54 (1.07–2.21).

**Table 2:** Allele/genotype frequencies of rs9939609 in study group

Genotype/allele	Frequency			OR (CI), *p*-value	
	Obese (*n* = 295)	CAD (*n* = 425)	Control (*n* = 250)	Obesity	CAD
TT	116	187	133	1.54 (1.07–2.21), 0.0009*	1.43 (1.02–2.05), 0.004*
TA	133	169	91		
AA	46	69	26		
T	0.621	0.637	0.716		
A	0.379	0.363	0.284		

The table shows the association of allele/genotype frequencies of rs9939609 in the cases and the control group. *CAD* Coronary Artery Disease group, *OR* Odds Ratio, *CI* Confidence Interval. *indicates any significant association

### Effect of FTO rs9939609 polymorphism on biochemical parameters

ANOVA was used to check whether the selected variant had any influence on serum biochemical traits (Table 3). The SNP did not appear to affect any of the tested lipid traits to a significant extent. A careful examination of the results revealed that the presence of the risk allele in homozygous state increased triglyceride level slightly when compared to TT or TA genotype in obese subjects and controls, whereas there is a decrease in HDL-C levels in all groups when the AA genotype is compared to TT or TA genotype although the effect is not statistically significant. However, the SNP appeared to be unambiguously associated with fasting blood sugar concentrations in CAD (*p* = 0. 014), obese (*p* = 0.001) and control (*p* = 0.019) groups.

**Table 3:** Effect of rs9939609 on biochemical traits in the study subjects

Group	Parameter	Genotype			*p*-value
		TT	TA	AA	
Controls (*n* = 250)	Total cholesterol (mmol/L)	4.38 ± 1.12	4.72 ± 1.36	4.67 ± 0.66	0.123
	Triglycerides (mmol/L)	2.34 ± 0.66	2.16 ± 0.75	2.73 ± 1.22	0.109
	HDLC (mmol/L)	1.80 ± 0.41	1.67 ± 0.15	1.65 ± 0.43	0.119
	LDLC (mmol/L)	2.13 ± 0.41	2.25 ± 0.48	2.26 ± 0.55	0.187
	FBG (mg/dL)	90.54 ± 6.81	90.81 ± 7.98	89.81 ± 5.41	0.019*
CAD (*n* = 425)	Total cholesterol (mmol/L)	5.41 ± 1.41	5.30 ± 1.36	5.38 ± 1.37	0.735
	Triglycerides (mmol/L)	2.35 ± 0.85	2.43 ± 0.81	2.41 ± 0.66	0.603
	HDLC (mmol/L)	1.15 ± 0.32	1.18 ± 0.29	1.16 ± 0.30	0.795
	LDLC (mmol/L)	2.73 ± 0.72	2.75 ± 0.73	2.71 ± 0.85	0.953
	FBG (mg/dL)	97.14 ± 6.81	98. + 1 ± 5.98	100.41 ± 6.41	0.014*
Obese (*n* = 295)	Total cholesterol (mmol/L)	5.50 ± 0.91	5.50 ± 0.74	5.49 ± 0.64	0.998
	Triglycerides (mmol/L)	2.51 ± 0.76	2.46 ± 0.75	2.71 ± 1.05	0.405
	HDLC (mmol/L)	1.11 ± 0.11	1.13 ± 0.11	1.07 ± 0.10	0.105
	LDLC (mmol/L)	2.96 ± 0.56	3.04 ± 0.54	2.95 ± 0.58	0.552
	FBG (mg/dL)	101.91 ± 13.52	104.21 ± 17.65	109.92 ± 16.54	0.001*

The table shows a comparison of mean biochemical parameters for three different genotypes in the study groups with strength of effect indicated by the *p*-value. *CAD* Coronary Artery Heart group, *HDL-C* High Density Lipoprotein cholesterol, *LDL-C* Low Density Lipoprotein cholesterol, *FBG* fasting blood glucose,*indicates any significant association

## Discussion

We have studied the association of *FTO* polymorphism rs9939609 with obesity and CAD in Pakistani population. Some *FTO* variants already associated with obesity are also associated with CAD risk factors (17). We proposed that since obesity is a well established risk factor for CAD, it is likely that the *FTO* gene, as the BMI/obesity related locus, might confer the risk of CAD. The SNP was found to be significantly associated with both obesity and CAD.

We found the frequency of risk allele (A) was higher in obese cases than controls. The risk allele of rs9939609 was significantly associated with the disease in Pakistani population. It has been reported that the presence of two alleles at the rs9939609 site of the *FTO* gene increased BMI by about 1 kg/m^2^, body mass by 2.3Kg and 1.3-fold higher risk of overweight and obesity in both adults and children. It has been estimated that per unit increase in BMI increase cardiovascular disease morbidity by 8 % [35]. We found similar results regarding association of the risk allele of rs9939609 with CAD in Pakistani subjects. It is the first study on this variant’s effect on CAD in Pakistan and successfully replicated the previous reports. However, the MAF was lower than reported in Caucasians consistent with a previous finding that the prevalence of the risk allele in East Asians (~20 %) and South Asians (~30 %) is substantially lower than in Europeans, and the reported effect sizes in both East and South Asians vary widely for BMI (OR 0.13–0.83 Kg/m^2^ per minor allele) and obesity risk (OR 1.02–1.48 per minor allele) [36]. Regarding the association between *FTO* gene and CAD risk, Doney et al first demonstrated that the A allele of rs9939609 increased the risk of myocardial infarction (MI) in 4897 patients with type 2 diabetes (T2D) in a prospective study, which was independent of BMI, glycohemoglobin, mean arterial pressure, HDL-C, triglycerides, and total cholesterol [34]. However, the subsequent studies revealed conflicting conclusions [31, 37–40]. A meta-analysis conducted in the end of 2013, comprising 19,153 cardiovascular disease (CVD) cases and 103,720 controls, pooled all these studies together, and found a significant association between the *FTO* gene variant rs9939609 and CVD risk independent of BMI and other conventional CVD risk factors [41].

The variant did not appear to influence any lipid parameter in the subjects tested, however, showed a consistent association with blood glucose levels. Therefore, it might play a role in progression to CAD through affecting plasma glucose metabolism. This is in accordance with a recent meta-analysis which reported that this variant has no correlation with serum lipid chemistry, and is associated with glucose levels [22]. However, the mechanism underlying the association of the *FTO* variant with CAD risk remains unclear yet. Previously, the rs9939609 variant was found to influence energy-dense food intake instead of regulation of energy expenditure [42]. In addition, it was also shown to be associated with diabetes-related metabolic traits (including higher fasting insulin, glucose and triglycerides, and lower HDL cholesterol), although the association disappeared after adjustment for BMI [43]. Other studies have indicated that *FTO* variant is associated with increased risk for hypertension through the regulation of sympathetic nervous system [44]. Notably, Hubacek et al believed that the *FTO* variant could increase the risk of CAD through another mechanism, mainly through its possible effect on DNA methylation. In other words, the *FTO* gene variant could interact with an unhealthy lifestyle (such as high fat diet and lack of physical activity), and affect the epigenetic status and ultimately contribute to the development of CVD [30].

The limitations of the study included a relatively small sample size and inability to include more biochemical and anthropometric measures in order to observe the real behaviour of the variant in the Pakistani ethnic group. The findings of the study should be therefore replicated in larger cohorts in future with more biochemical parameter included in order to validate the results observed in the current investigation and find out new associations, if any, of the variant.

In conclusion, we have shown that the *FTO* variant rs9939609 is associated with obesity and showed for the first time in Pakistan, with coronary artery disease. We confirmed the association of this variant with plasma glucose, but couldn’t detect any effect on serum lipid traits.

## Methods

### Study subjects

A total of 970 subjects were included in the study after obtaining informed consent. The study groups consisted of 295 obese individuals (43.8 % females, 56.2 % males, mean age 39.21 ± 15.18), 425 CAD patients (41 % females, 59 % males, mean age 59 ± 12) and 250 controls (45.7 % females, 54.3 % males, mean age 56 ± 10). The obese subjects were recruited based on BMI and waist to hip ratio (WHR). The BMI cutoffs used were those defined for the Asian population and have been described elsewhere [45]. An additional measure to differentiate between obese and non obese used was WHR and a value of ≥ 0.85 in females and ≥ 1 in males was used as cutoff [46]. The CAD patients were selected from tertiary care hospitals of the province of Punjab, Pakistan. These were diagnosed cases based upon the ECG, cardiac echo, radiologic and troponine T/I levels. These cases were recently diagnosed of having CAD and were not taking any lipid lowering or antihypertensive drug. Control subjects were apparently healthy individuals from the general population with BMI 18.5–22.99 Kg/m^2^ and no history of heart disease. The exclusion criteria included the presence of any chronic ailment like chronic liver or kidney disease, cancers or any ongoing acute infection or disease and CAD patients with obesity.

### Ethics, consent and permissions

All the procedures were in compliance with the Helsinki Declaration and the study was approved by the institutional ethics committee.

### Blood sample collection

Early morning blood samples were collected from the median cubital vein taking aseptic measures. The blood samples were collected into two vacutainers (BD, USA), one coated with EDTA as an anticoagulant and the other containing a gel and clot activator at the bottom. The whole blood sample from EDTA tube was used for DNA extraction, while the clotted blood in gel vials was centrifuged at 5,000rpm for 5min to separate serum. The serum samples were used for determining biochemical parameters.

### Determination of biochemical parameters

The serum samples were analyzed for total cholesterol (TC), high density lipoprotein cholesterol (HDL-C) and triglycerides (TG) levels. The end point spectrophotometric methods were adopted according to the manufacturers’ guidelines using commercially available kits (Human Diagnostics, Germany). LDL-C was measured by Friedwalds equation. Epoch, Biotek microplate reader (Biotek instruments, Highland Park) was used for all optical density measurements. Fasting blood glucose (FBG) was measured using a digital glucometer (Sky-ERA, TD 4103).

### Genotyping

Genomic DNA was isolated from peripheral blood leukocytes using Promega Wizard® Genomic DNA purification kit. DNA was quantified using nanodrop (ND-8000, USA). The DNA samples were diluted to a standard concentration of 1.25ng/μl. A 4μl of this dilution was taken to get the final working concentration of 5ng of DNA. A liquid handling robotic system (Biomerk FX, Beckman Coulter) was used to spot DNA in 384-well plates (Micro Amp), specially designed for PCR amplification. Genotyping was done by TaqMan allelic discrimination assay using primers and probes given in Additional file 1: Table S1. The real time PCR reaction mixture consisted of 1X KAPA probe fast qPCR master mix (KAPABiosystems, USA), 100nM of each primer, 100nM of each probe, ROX high (0.4μl/20 μl reaction mixture), 5ng DNA and PCR grade water as needed. The PCR program consisted of an initial temperature of 50 °C for 2 min Then denaturation/enzyme activation at 95 °C for 10 min, finally amplification for 40 cycles each consisting of denaturation at 95 °C for 15s and amplification at 60 °C for1 min. PCR was done on the Bird C1000TM thermal cycler. After amplification the results were analyzed on the ABI Prism 7900HT (Applied Biosystems/Life Technologies) and the genotypes were called using sequence detection software (SDS) version 2.0.

### Statistical analysis

Statistical Analysis was done using Statistical Package for Social Sciences (SPSS, IBM statistics, version 22). Independent sample *t*-test was used to compare the anthropometric and biochemical parameters of obese and CHD groups with the controls. The study population was tested for Hardy Weinberg equilibrium and allele/genotype frequencies were calculated. The significance of difference in allele and genotype frequencies was calculated using a chi-squared test. The odds ratio was calculated using binary logistic regression. One-way analysis of variance (ANOVA) was used to check the effect of rs9939609 polymorphism on biochemical parameters. A *p*-value <0.05 was considered statistically significant for all analyses.

## Additional file

Additional file 1: Table S1. Primers and Probes for TaqMan Assay. (DOCX 10 kb)

## References

[1] Bell CG, Walley AJ, Froguel P (2005). The genetics of human obesity. Nat Re Genes.

[2] Shabana, Hasnain S (2015). Association of the leptin receptor Gln223Arg polymorphism with lipid profile in obese Pakistani subjects. Nutrition.

[3] Day FR, Loos RJ (2011). Developments in obesity genetics in the era of genome-wide association studies. J Nutrigenet Nutrigenom.

[4] Hinney A, Vogel CI, Hebebrand J (2010). From monogenic to polygenic obesity: recent advances. Eur Child Adolesc Psychiatry.

[5] Abraham G, Bhalala OG, de Bakker PI, Ripatti S, Inouye M (2014). Towards a molecular systems model of coronary artery disease. Cur Cardiol Rep.

[6] Joshi P, Islam S, Pais P, Reddy S, Dorairaj P, Kazmi K, Pandey MR, Haque S, Mendis S, Rangarajan S. Risk factors for early myocardial infarction in South Asians compared with individuals in other countries. JAMA. 2007;297:286–94. 10.1001/jama.297.3.286 17227980

[7] Saleheen D, Soranzo N, Rasheed A, Scharnagl H, Gwilliam R, Alexander M, Inouye M, Zaidi M, Potter S, Haycock P. Genetic determinants of major blood lipids in Pakistanis compared with Europeans. Circ Cardiovas Genet. 2010;3:348–57. 10.1161/CIRCGENETICS.109.906180 20570915

[8] Nuri M, Nawaz A, Usman H: A proposed study of risk factors of heart disease in rural population of punjab (pakistan)—time to act! *Pak Heart J*. 2012;39:42-7.

[9] Flegal KM, Graubard BI, Williamson DF, Gail MH (2008). Cause-Specific Excess Deaths Associated With Underweight, Overweight, and Obesity. Obstet Gynecol Surv.

[10] Peng S, Zhu Y, Xu F, Ren X, Li X, Lai M (2011). FTO gene polymorphisms and obesity risk: a meta-analysis. BMC Med.

[11] Loos R, Bouchard C (2008). FTO: the first gene contributing to common forms of human obesity. Obes Revi.

[12] Robbens S, Rouzé P, Cock JM, Spring J, Worden AZ, Van de Peer Y (2008). The FTO gene, implicated in human obesity, is found only in vertebrates and marine algae. J Mol Evol.

[13] Gerken T, Girard CA, Tung Y-CL, Webby CJ, Saudek V, Hewitson KS, Yeo GS, McDonough MA, Cunliffe S, McNeill LA. The obesity-associated FTO gene encodes a 2-oxoglutarate-dependent nucleic acid demethylase. Science. 2007;318:1469–72. 10.1126/science.1151710 PMC2668859 17991826

[14] Fredriksson R, Hagglund M, Olszewski PK, Stephansson O, Jacobsson JA, Olszewska AM, Levine AS, Lindblom J, Schioth HB. The obesity gene, FTO, is of ancient origin, up-regulated during food deprivation and expressed in neurons of feeding-related nuclei of the brain. Endocrinology. 2008;149:2062–71. 10.1210/en.2007-1457 18218688

[15] Price RA, Li W-D, Zhao H (2008). FTO gene SNPs associated with extreme obesity in cases, controls and extremely discordant sister pairs. BMC Med Genet.

[16] Peeters A, Beckers S, Verrijken A, Roevens P, Peeters P, Van Gaal L, Van Hul W. Variants in the FTO gene are associated with common obesity in the Belgian population. Mol Genet Metabol. 2008;93:481–4. 10.1016/j.ymgme.2007.10.011 18055244

[17] Grant SF, Li M, Bradfield JP, Kim CE, Annaiah K, Santa E, Glessner JT, Casalunovo T, Frackelton EC, Otieno FG. Association analysis of the FTO gene with obesity in children of Caucasian and African ancestry reveals a common tagging SNP. PLoS One. 2008;3:e1746. 10.1371/journal.pone.0001746 PMC2262153 18335027

[18] Li H, Wu Y, Loos RJ, Hu FB, Liu Y, Wang J, Yu Z, Lin X. Variants in the fat mass–and obesity-associated (FTO) gene are not associated with obesity in a Chinese Han population. Diabetes. 2008;57:264–8. 10.2337/db07-1130 17959933

[19] Hennig BJ, Fulford AJ, Sirugo G, Rayco-Solon P, Hattersley AT, Frayling TM, Prentice AM. FTO gene variation and measures of body mass in an African population. BMC Med Genet. 2009;10:21. 10.1186/1471-2350-10-21 PMC2666669 19265514

[20] Fang H, Li Y, Du S, Hu X, Zhang Q, Liu A, Ma G. Variant rs9939609 in the FTO gene is associated with body mass index among Chinese children. BMC Med Genet. 2010;11:136. 10.1186/1471-2350-11-136 PMC2955568 20858286

[21] Cha SW, Choi SM, Kim KS, Park BL, Kim JR, Kim JY, Shin HD. Replication of genetic effects of FTO polymorphisms on BMI in a Korean population. Obesity. 2008;16:2187–9. 10.1038/oby.2008.314 18551112

[22] Liu Y, Liu Z, Song Y, Zhou D, Zhang D, Zhao T, Chen Z, Yu L, Yang Y, Feng G. Meta‐analysis added power to identify variants in FTO associated with type 2 diabetes and obesity in the Asian population. Obesity. 2010;18:1619–24. 10.1038/oby.2009.469 20057365

[23] Karasawa S, Daimon M, Sasaki S, Toriyama S, Oizumi T, Susa S, Kameda W, Wada K, Muramatsu M, Fukao A. Association of the common fat mass and obesity associated (FTO) gene polymorphism with obesity in a Japanese population. Endocrine J. 2010;57:293–301. 10.1507/endocrj.k09e-305 20051647

[24] Hotta K, Nakata Y, Matsuo T, Kamohara S, Kotani K, Komatsu R, Itoh N, Mineo I, Wada J, Masuzaki H. Variations in the FTO gene are associated with severe obesity in the Japanese. J Hum Genet. 2008;53:546–53. 10.1007/s10038-008-0283-1 PMC2413114 18379722

[25] Tan JT, Dorajoo R, Seielstad M, Sim XL, Ong RT-H, Chia KS, Wong TY, Saw SM, Chew SK, Aung T. FTO variants are associated with obesity in the Chinese and Malay populations in Singapore. Diabetes. 2008;57:2851–7. 10.2337/db08-0214 PMC2551698 18599522

[26] Dorajoo R, Blakemore A, Sim X, Ong RT, Ng D, Seielstad M, Wong T, Saw S, Froguel P, Liu J. Replication of 13 obesity loci among Singaporean Chinese, Malay and Asian-Indian populations. Int J Obes. 2012;36:159–63. 10.1038/ijo.2011.86 21544081

[27] Ramya K, Radha V, Ghosh S, Majumder PP, Mohan V (2011). Genetic variations in the FTO gene are associated with type 2 diabetes and obesity in south Indians (CURES-79). Diab Technol Therap.

[28] Yajnik CS, Janipalli CS, Bhaskar S, Kulkarni SR, Freathy RM, Prakash S, Mani KR, Weedon MN, Kale SD, Deshpande J. FTO gene variants are strongly associated with type 2 diabetes in South Asian Indians. Diabetologia. 2009;52:247–52. 10.1007/s00125-008-1186-6 PMC2658005 19005641

[29] He M, Cornelis MC, Franks PW, Zhang C, Hu FB, Qi L (2010). Obesity genotype score and cardiovascular risk in women with type 2 diabetes mellitus. Arterioscler Thromb Vasc Biol.

[30] Hubacek JA, Staněk V, Gebauerová M, Pilipčincová A, Dlouhá D, Poledne R, Aschermann M, Skalická H, Matoušková J, Kruger A. A FTO variant and risk of acute coronary syndrome. Clin Chim Acta. 2010;411:1069–72. 10.1016/j.cca.2010.03.037 20362563

[31] Lappalainen T, Kolehmainen M, Schwab U, Tolppanen A, Stančáková A, Lindström J, Eriksson J, Keinänen-Kiukaanniemi S, Aunola S, Ilanne-Parikka P. Association of the FTO gene variant (rs9939609) with cardiovascular disease in men with abnormal glucose metabolism–The Finnish Diabetes Prevention Study. Nut Metabol Cardiovas Dis. 2011;21:691–8. 10.1016/j.numecd.2010.01.006 20400278

[32] Nguyen NT, Magno CP, Lane KT, Hinojosa MW, Lane JS (2008). Association of hypertension, diabetes, dyslipidemia, and metabolic syndrome with obesity: findings from the National Health and Nutrition Examination Survey, 1999 to 2004. J Am Coll Surg.

[33] Scuteri A, Sanna S, Chen W-M, Uda M, Albai G, Strait J, Najjar S, Nagaraja R, Orrú M, Usala G. Genome-wide association scan shows genetic variants in the FTO gene are associated with obesity-related traits. PLoS Genet. 2007;3:e115. 10.1371/journal.pgen.0030115 PMC1934391 17658951

[34] Doney AS, Dannfald J, Kimber CH, Donnelly LA, Pearson E, Morris AD, Palmer CN. The FTO Gene Is Associated With an Atherogenic Lipid Profile and Myocardial Infarction in Patients With Type 2 Diabetes A Genetics of Diabetes Audit and Research Study in Tayside Scotland (Go-DARTS) Study. Circ Cardiovas Genet. 2009;2:255–9. 10.1161/CIRCGENETICS.108.822320 PMC3045745 20031593

[35] Li TY, Rana JS, Manson JE, Willett WC, Stampfer MJ, Colditz GA, Rexrode KM, Hu FB. Obesity as compared with physical activity in predicting risk of coronary heart disease in women. Circulation. 2006;113:499–506. 10.1161/CIRCULATIONAHA.105.574087 PMC3210835 16449729

[36] Li H, Kilpeläinen T, Liu C, Zhu J, Liu Y, Hu C, Yang Z, Zhang W, Bao W, Cha S. Association of genetic variation in FTO with risk of obesity and type 2 diabetes with data from 96,551 East and South Asians. Diabetologia. 2012;55:981–95. 10.1007/s00125-011-2370-7 PMC3296006 22109280

[37] Winter Y, Back T, Scherag A, Linseisen J, Rohrmann S, Lanczik O, Hinney A, Scherag S, Neumaier M, Ringleb PA. Evaluation of the obesity genes FTO and MC4R and the type 2 diabetes mellitus gene TCF7L2 for contribution to stroke risk: The Mannheim-Heidelberg Stroke Study. Obesity Facts. 2011;4:290–6. 10.1159/000330881 PMC6444624 21921652

[38] Berzuini C, Vansteelandt S, Foco L, Pastorino R, Bernardinelli L (2012). Direct Genetic Effects and Their Estimation From Matched Case‐Control Data. Genet Epidemiol.

[39] Borglykke A, Grarup N, Sparsø T, Linneberg A, Fenger M, Jeppesen J, Hansen T, Pedersen O, Jørgensen T: Genetic Variant SCL2A2 Is Associated with Risk of Cardiovascular Disease–Assessing the Individual and Cumulative Effect of 46 Type 2 Diabetes Related Genetic Variants. *Plos One*. 2012;7:1-8. 10.1371/journal.pone.0050418 PMC3503928 23185617

[40] Nordestgaard BG, Palmer TM, Benn M, Zacho J, Tybjærg-Hansen A, Davey Smith G, Timpson NJ. The effect of elevated body mass index on ischemic heart disease risk: causal estimates from a Mendelian randomisation approach. PLoS Med. 2012;9:628. 10.1371/journal.pmed.1001212 PMC3341326 22563304

[41] Liu C, Mou S, Pan C (2013). The FTO gene rs9939609 polymorphism predicts risk of cardiovascular disease: a systematic review and meta-analysis. PLoS One.

[42] Razquin C, Marti A, Martinez JA (2011). Evidences on three relevant obesogenes: MC4R, FTO and PPARγ. Approaches for personalized nutrition. Mol Nut Food Res.

[43] Freathy RM, Timpson NJ, Lawlor DA, Pouta A, Ben-Shlomo Y, Ruokonen A, Ebrahim S, Shields B, Zeggini E, Weedon MN. Common variation in the FTO gene alters diabetes-related metabolic traits to the extent expected given its effect on BMI. Diabetes. 2008;57:1419–26. 10.2337/db07-1466 PMC3073395 18346983

[44] Pausova Z, Syme C, Abrahamowicz M, Xiao Y, Leonard GT, Perron M, Richer L, Veillette S, Smith GD, Seda O. A common variant of the FTO gene is associated with not only increased adiposity but also elevated blood pressure in French Canadians. Circ Cardiovas Genet. 2009;2:260–9. 10.1161/CIRCGENETICS.109.857359 20031594

[45] Shabana, Shahid SU, Li KW, Acharya J, Cooper J, Hasnain S, Humphries SE: Effect of six type 2 diabetes susceptibility loci and an FTO variant on Obesity in Pakistani subjects. *Eur J Hum Genet* 2015, In Press. doi: 10.1038/ejhg.2015.212. 10.1038/ejhg.2015.212 PMC4867451 26395551

[46] Yusuf S, Hawken S, Ôunpuu S, Bautista L, Franzosi MG, Commerford P, Lang CC, Rumboldt Z, Onen CL, Lisheng L. Obesity and the risk of myocardial infarction in 27 000 participants from 52 countries: a case-control study. Lancet. 2005;366:1640–9. 10.1016/S0140-6736(05)67663-5 16271645

