# Supplementary material for: Role of a common variant of Fat Mass and Obesity associated (FTO) gene in obesity and coronary artery disease in subjects from Punjab, Pakistan: a case control study
Source: Lipids Health Dis. 2016 Feb 16;15:29. doi: 10.1186/s12944-016-0200-0 (PMC4754878; doi:10.1186/s12944-016-0200-0)
Supplement: Additional file 1: Table S1. — Primers and Probes for TaqMan Assay. (DOCX 10 kb) [file 12944_2016_200_MOESM1_ESM.docx]

| SNP | Primers | TaqMan Probes | Dye (position 8) |
| --- | --- | --- | --- |
| rs9939609 | ACTAACATCAGTTATGCATTTAGAATGTCTGA | TGCATCACAAAATTC | VIC |
|  | ACCTATTAAAACTTTAGAGTAACAGAGACTATCCA | TGCATCACTAAATTC | FAM |

Additional file 1: Table S1: Primers and Probes for TaqMan Assay
